# Structure Prediction of the Second Extracellular Loop in G-Protein-Coupled Receptors

**DOI:** 10.1016/j.bpj.2014.04.022

**Published:** 2014-06-03

**Authors:** Sebastian Kmiecik, Michal Jamroz, Michal Kolinski

**Affiliations:** † University of Warsaw, Faculty of Chemistry, Laboratory of Theory of Biopolymers, Pasteura 1, 02-093 Warsaw, Poland; ‡ Mossakowski Medical Research Center, Polish Academy of Sciences, Bioinformatics Laboratory, Pawinskiego 5, 02-106 Warsaw, Poland

## Abstract

G-protein-coupled receptors (GPCRs) play key roles in living organisms. Therefore, it is important to determine their functional structures. The second extracellular loop (ECL2) is a functionally important region of GPCRs, which poses significant challenge for computational structure prediction methods. In this work, we evaluated CABS, a well-established protein modeling tool for predicting ECL2 structure in 13 GPCRs. The ECL2s (with between 13 and 34 residues) are predicted in an environment of other extracellular loops being fully flexible and the transmembrane domain fixed in its x-ray conformation. The modeling procedure used theoretical predictions of ECL2 secondary structure and experimental constraints on disulfide bridges. Our approach yielded ensembles of low-energy conformers and the most populated conformers that contained models close to the available x-ray structures. The level of similarity between the predicted models and x-ray structures is comparable to that of other state-of-the-art computational methods. Our results extend other studies by including newly crystallized GPCRs.

## Introduction

G-protein-coupled receptors (GPCRs) constitute the largest and the most versatile family of membrane-bound receptors. They interact with very diverse sets of ligands including neurotransmitters, hormones, amino acids, lipids, odorants, ions, fatty acids, and peptides. In response to stimuli, the receptor undergoes a series of structural rearrangements (1) allowing signal transduction across the plasma membrane and its further propagation inside the cell. Because GPCRs play key roles in a variety of signaling cascades that control many cellular processes and are related to numerous diseases (2), they are very important targets for pharmacological intervention (3). It is estimated that ∼40% of drugs currently in clinical use target these receptor proteins (4,5). Significant effort is devoted to determine human GPCR structures and function (6), which may lead to the discovery of new potent drugs with higher receptor subtype selectivity (and thus fewer side effects). Thanks to the recent progress in crystallization techniques, structural coverage of GPCRs has experienced an exponential growth trend (6). However, the gap between the number of experimentally derived crystal structures and all known GPCR sequences (potential new drug targets) remains large (sequences of >800 GPCRs are now identified (7)). This makes computational methods a reasonable and promising alternative for the determination of receptor atomic structures.

All GPCRs share a common architecture of a seven-helix bundle spanning the cell membrane. This region shows the highest sequential conservation among all members of the GPCR family. The seven transmembrane helices (TMHs) are linked by intra- and extracellular loop regions. The loop regions present significant structural diversity even between closely related receptor subtypes (8). The most interesting GPCR region for structure-based drug design is the ligand interaction and recognition site located in the cavity created by surrounding TMHs and extracellular loops (ECLs). Over the last decade, the ECLs have gained increasing interest due to their important functional roles in ligand binding, activation, and regulation of GPCRs (9). The accurate prediction of ECLs is critical for the construction of models applicable in drug design efforts (8,9). The low sequence similarity and lack of suitable templates makes homology modeling methods inappropriate for this purpose. Different computational protocols have been applied to the prediction of ECL structures in different GPCRs (10–14). Most of them showed that short ECLs (5–7 residues) can be predicted with very good accuracy (with root mean-square deviations (RMSDs) lower than 1 Å when compared to the crystal structures). In contrast, the prediction of long (or so called super-long ECLs, having over 15 residues) presents a challenging task for contemporary modeling tools.

The second ECL (ECL2) that connects TMH4 and TMH5 is the longest and the most divergent of the three ECLs. The functional importance of ECL2 has been demonstrated in many studies. For instance, ECL2 has been shown to play an important role in binding both allosteric and orthosteric ligands (8), receptor function and signaling (15,16). Mutagenesis studies also confirmed that the ECL2 region is responsible for the receptor subtype selectivity of signaling molecules (17,18) and its alteration may transform an antagonist to act as an agonist (19). Moreover, a particular ECL2 conformation is probably required for preserving proper receptor-ligand interaction, e.g., disruption of the disulfide bond stabilizing the short *α*-helix present in ECL2 of the adrenergic receptor decreased ligand affinity 1000-fold (20). In addition, long scale molecular dynamics (MD) simulation of the adrenergic receptor suggested that the ECL2 region is responsible for preliminary interaction with small molecules entering the binding site (21). The importance of ECL2 for receptor activation was also highlighted by the identification of point mutations conferring constitutive activity of the C5a receptor (22) and the thrombin receptor (23).

In this work, we present results of ECL2 structure prediction for 13 subtypes of GPCRs (representing all receptor subtypes with available crystal structures at a time when this study was initiated). The following receptors were selected for ECL2 restoration: Adenosine receptor A2a (A2AR), Beta-1 adrenergic receptor (*β*1AR), Beta-2 adrenergic receptor (*β*2AR), C-X-C chemokine receptor type 4 (CXCR4), Dopamine D3 receptor (D3R), Delta-type opioid receptor (DOR), Muscarinic acetylcholine receptor M2 (M2R), Muscarinic acetylcholine receptor M3 (M3R), Mu-type opioid receptor (MOR), Nociceptin receptor (NOP), Neurotensin receptor type 1 (NTR1), Rhodopsin (RHO), and Sphingosine 1-phosphate receptor 1 (S1PR). For each receptor, we chose one crystal structure from the Protein Data Bank (PDB) database showing the highest resolution and complete representation of extracellular loops (see Table 1 for receptor details). Of importance, in our modeling we used no information about the crystal structure of any extracellular element (including ECL1, ECL2, and ECL3), except constraints on disulfide bridges.

## Methods

**Figure 1:**
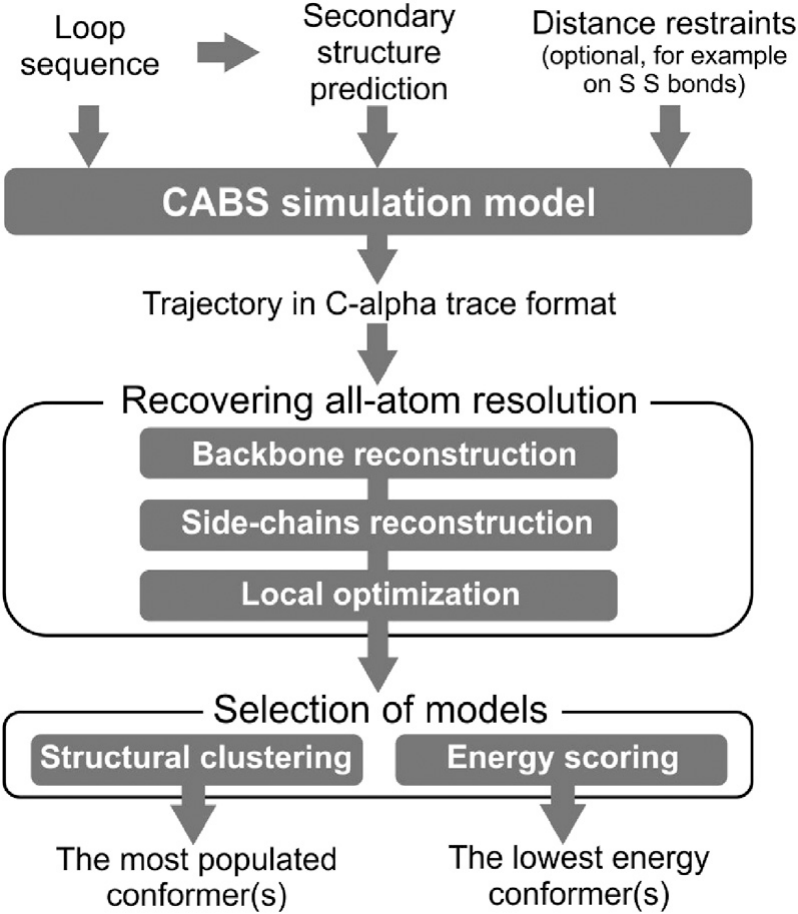
Pipeline of the loop modeling procedure.

In Fig. 1, we present a pipeline of the loop modeling procedure employed in this work. The procedure consists of three major modeling steps: 1), exploring the conformational space by the CABS model; 2), reconstruction to all-atom representation; and 3), selection of resulting model(s).

### CABS model

CABS (C-Alpha, Beta, and Side chain) is a versatile protein modeling tool based on coarse-grained structure representations and the Monte Carlo dynamics sampling scheme. CABS has been extensively tested in numerous structure prediction exercises, including successful participation in CASP experiments (CASP, Critical Assessment of protein Structure Prediction, a community-wide blind test of structure prediction approaches). In the CASP6 edition the Kolinski-Bujnicki group, employing the CABS-based modeling strategy, scored as the best, or the second best, depending on the evaluation method (24,25). The CABS modeling tool was also productive in the ab initio prediction of protein loops (26) or missing fragments (27), high-resolution structure prediction (28), modeling of protein-protein complexes (29,30), or large biomolecular systems (31,32). Taken together, those tests demonstrated that the CABS approach is competitive, or even superior, to other state-of-the-art structure prediction tools especially in difficult modeling cases (typically when large protein fragments need to be predicted with little or no support from evolutionary or experimental data). Recently, the CABS approach for the ab initio and consensus-based prediction of protein structure has been made available as a CABS-fold web server (33).

The CABS model is described in detail elsewhere (34). Here, we give only a brief summary. The major components of the CABS model (protein representation, force field, and sampling scheme) have been designed for the efficient simulation of real proteins. The CABS protein representation has been reduced to up to four atoms per residue: alpha carbon, beta carbon, and two pseudoatoms: center of mass of the side chain and center of the virtual alpha carbon-alpha carbon bond. The CABS force field employs knowledge-based potentials derived from statistical analysis of known protein structures (deposited in the PDB) and a model of main-chain hydrogen bonds. Solvent effects are accounted for in an implicit way through the knowledge-based potentials. The CABS dynamics is simulated by a random series of local micromodifications controlled by the asymmetric Metropolis scheme of the Monte Carlo method. Of importance, the long series of such micromodifications describes well near-native dynamics (35,36) or entire protein folding mechanisms (37–39). Detailed analysis of CABS dynamics, together with its comparison to MD simulation and other computational tools, is provided in the work (36).

The resolution of CABS predictions enables fast reconstruction to realistic all-atom models. Thus, the CABS model can be easily merged with all-atom modeling tools into multiscale modeling procedures benefiting from coarse-grained efficiency and atomic-level accuracy (40,41).

### CABS setup and modifications for the present study

The required CABS input files were prepared using the Bioshell package (42). CABS simulations started from random conformations of EC loops. TM fragments of receptor structures were restrained to x-ray structure (using distance restraints on alpha carbons). For each receptor, two independent CABS runs were conducted, each generating 2000 models. Therefore, in total, 4000 CABS-generated models for each receptor were used in the next modeling steps.

The CABS model performs very well for a large fraction of globular proteins (24), but the statistical potential needs some corrections for specific systems. In the generic force field the CYS-CYS side chain contact potential reflects the statistical average for bonded and unbonded pairs (34). For the systems studied in this work we assume knowledge of bonded CYS pairs, the CYS-CYS statistical potential has been assumed to be 0, whereas on the bonded pairs we imposed strong distance restrains. This way possible artificial energy biases toward the more than binary CYS contacts have been eliminated.

In the original force field of CABS the interaction distance of side chains was derived for single domain globular proteins. In this application we slightly reduced the effective width and stiffness of amino acids from loop-forming sequences (d1 = 0.5 and d2 = 1.5, see a detailed description of the original force field in (34).). This way, we perhaps slightly decreased the accuracy of the discrete representation of low energy folded structures, enabling, however, efficient transitions between various local minima.

### Reconstruction to all-atom representation and selection of model(s)

In general, the reconstruction to all-atom representation involved a three step procedure: i), reconstruction of the backbone chain based on the alpha-carbon trace; ii), reconstruction of side-chain positions based on the backbone chain; iii), short optimization and refinement protocol. In more detail, in the first step CABS-generated trajectories (in the C-alpha format) were reconstructed to backbone representation using the BBQ tool (43). The prepared loop conformations were inserted into the native crystal structure, and loop side chains were reconstructed with SCWRL3 (44). In the next step, each model was optimized with the DOPE force field (45) using MODELER by a comparative modeling procedure (using previous step models as templates). Loop side chains were again optimized with SCWRL3 (44). The constructed models were subjected to energy calculation and structural clustering. All-atom energy was evaluated with GROMACS software (46) using single point energy computation. Structural clustering was performed with the K-means algorithm (using ClusCo software (47)).

RMSDs of loops were calculated using CSB (48) on loop fragments, after superimposition of the whole model onto the native/reference structure (excluding loop atoms).

### Selection of ECLs

The ECL fragment boundaries were selected based on examination of the secondary structure of TM domains in receptor crystal structures (x-ray structures are listed in Table 1). The first and the last amino acid of the ECLs were considered as the one not involved in the TM helices hydrogen bond network. Table 2 lists sequences of the ECLs restored in this study for 13 GPCRs.

### Secondary structure prediction

The CABS modeling procedure can be supported with additional information about the expected types of secondary structures. CABS uses different sets of statistical potentials for protein fragments with assigned secondary structure (three predefined potential types are available: H for *α*-helical conformations, E for extended conformations, and C for coil-like conformations). These different sets of potentials are mainly responsible for controlling distances between respective alpha carbons (C*α*_n_ - C*α*_n+2_ and C*α*_n_ - C*α*_n+4_ pairs, for a detailed description of the CABS force field see (34)). Therefore, to enhance the accuracy of final predictions, we enriched the input data by theoretical predictions of ECL2 secondary structure. The input predictions were obtained as a consensus from three web server tools (predicting secondary structure from sequence): PSIPRED (49), Jpred 3 (50), and PSSpred (51) (see Table S2 in the Supporting Material for consensus secondary structure prediction). In our experience, a correct secondary structure input can significantly improve prediction results, although input mistakes can have serious consequences on the final outcome (input overpredictions of the regular secondary structure are more dangerous for the quality of the results than underpredictions (52,53)).

## Results

### Comparison of modeling results with experimental crystal structures

**Figure 2:**
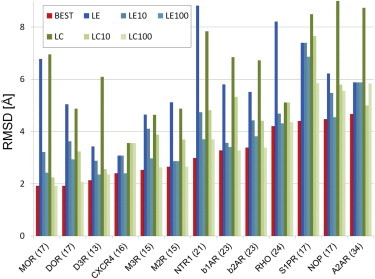
Results of predictions of the second extracellular loop (ECL2). GPCR receptors (with ECL2 residue length in brackets) are marked on the horizontal axis. For each receptor, the color bars show RMSD (in Å from crystal structures) of models selected according to different criteria. Red bars correspond to the lowest RMSD model generated by CABS. Blue shadow bars correspond to models selected based on energy scores: model with the lowest energy (LE), model showing the lowest RMSD from the 10 lowest energy models (LE10) and model showing the lowest RMSD from the 100 lowest energy models (LE100). Green shadow bars correspond to models selected based on structural clustering: model representing the largest cluster (LC), model showing the lowest RMSD from the representatives of 10 largest clusters (LC10), model showing the lowest RMSD from the representatives of 100 largest clusters (LC100). The detailed values are given in Table S3 and Table S4. To see this figure in color, go online.

In Fig. 2, we present a summary of structure prediction results of ECL2 loops (for details of the modeling procedure, see Methods). The figure shows the lowest RMSD values obtained by the CABS model (*red bars*) and RMSDs of CABS-generated models selected according to all-atom energy values (*blue shadowed bars*), and structural clustering (*green shadowed bars*). The results of the selection are presented for a single top-scored model (the lowest energy one, LE; or representing the largest cluster, LC), but also for the lowest RMSD models observed within a set of top-scored models (10 or 100). According to Nikiforovich et al. (12,54), the lowest RMSD out of a set of top-scored models may be a more adequate measure of prediction accuracy than RMSD of a single top-scored model (12,54). This is because crystal structures capture single conformation only of highly mobile ECL loops, but not necessarily the most biologically relevant one. Therefore, in Fig. 2 we report the lowest RMSD values observed within the sets of 10 or 100 of the lowest energy models (LE10 or LE100) and the sets of 10 or 100 representatives of the largest clusters (LC10 or LC100).

As presented in Fig. 2, the best RMSD models obtained by CABS are within an RMSD range of 1.9–4.7 Å from their crystal structures (depending on GPCR). These models represent the lowest RMSD value (RMSD^BEST^) observed in a trajectory of 4000 snapshots generated by CABS for each GPCR target. As already mentioned previously, we attempted to reduce the number of alternative predictions (from 4000 to 1 or 10 or 100) using well-tested selection procedures: all-atom energy scoring after short minimization in the all-atom force-field (28) and structural clustering (24,26) (see Methods for details).

We applied an energy scoring and minimization procedure similar to the one that proved efficient in the discrimination of medium-accuracy homology models (RMSD range of 2–3 Å from the native) from low-accuracy homology models of globular proteins (see Fig. 4 in (28).). Analysis of RMSDs of the lowest energy models (RMSD^LE^) shows that in most GPCR cases RMSD^LE^ values are disappointingly higher than corresponding RMSD^BEST^ values. Taken together this indicates that the energy evaluation of GPCR loops is a more demanding task than that of homology models of globular proteins in (28). On the other hand, the values of the lowest RMSDs from the set of 10 or 100 selected models (see RMSD^LE10^ and RMSD^LE100^ in Fig. 2) are in most receptor cases close to the RMSD^BEST^ values.

In addition to energy scoring, we applied structural clustering as an alternative approach to model selection. Using a clustering method, which proved to be useful in previous structure prediction tasks (24,26), we attempted to select a single representative model and sets of models (10 or 100, similarly as by energy scoring). As shown in Fig. 2, in most GPCR cases representative models of the largest cluster have substantially higher RMSD values (RMSD^LC^) than RMSD^BEST^. However, in two GPCR cases (CXCR4 and RHO) the representatives of the largest cluster have the lowest RMSD among the representatives of the 10 largest clusters (for CXCR4 RMSD^LC^ = RMSD^LC10^ = 3.56 Å, and for RHO RMSD^LC^ = RMSD^LC10^ = 5.11 Å, see Fig. 2). In summary, results of the selection of models using structural clustering were on average comparable (slightly inferior) to those of energy scoring. Namely the average RMSD values (for the entire GPCR set) were the following: RMSD^BEST^ = 3.15 Å, RMSD^LE^ = 5.84 Å, RMSD^LE10^ = 4.3 Å, RMSD^LE100^ = 3.73 Å, RMSD^LC^ = 6.47 Å, RMSD^LC10^ = 4.41 Å and RMSD^LC100^ = 3.63 Å (for the description of RMSD superscripts see Fig. 2, *legend*). All the predicted models are available for download from http://biocomp.chem.uw.edu.pl/GPCR-loop-modeling/).

The model evaluation presented previously was based on comparison with the highest resolution x-ray structure of each receptor subtype (see Table 1). Furthermore, we extended the comparison to all additional crystal structures of each GPCR subtype when available (from the GPCRSD database (55), see their list in Table S5). Calculated RMSD values showed no qualitative differences from those reported previously (see Table S6). In addition, we estimated the conservation of ECL2 structure among all available x-ray structures using previously chosen highest resolution structures as reference structures (see Table S5). The highest RMSD value = 2.5 Å was observed for M2R with a bound agonist, whereas most of the analyzed structures showed very low RMSD values < 1 Å. Furthermore, visual inspection of superimposed x-ray structures indicated very small differences in ECL2 conformation among the same receptor subtypes.

### Analysis of example models

**Figure 3:**
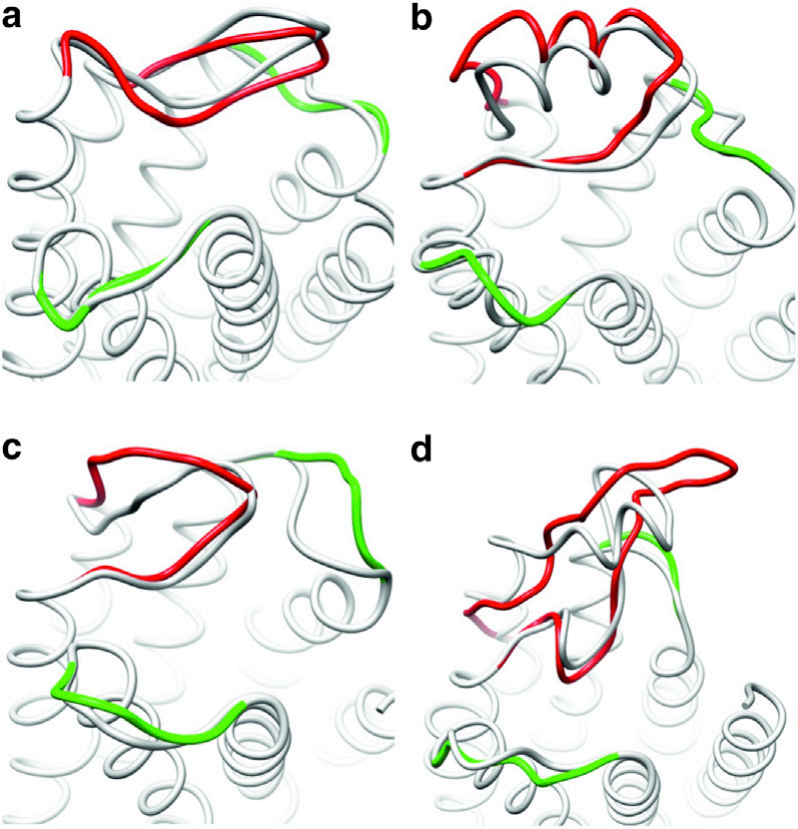
Example predictions superimposed on crystal structures. Predicted models of the second extracellular loop are shown in red, together with the first and third extracellular loops colored in green, and the reference crystal structure shown in gray. The following models are presented: (*a*) CXCR4 model, representative of the largest cluster (RMSD^LC^ = 3.56 Å), (*b*) *β*1AR model, from the set of 10 lowest energy models (RMSD^LE10^=3.57 Å), (*c*) D3R model, from the set of 10 lowest energy models (RMSD^LE10^=2.88 Å), (*d*) A2AR model, from the set of 10 lowest energy models (RMSD^LE10^=5.88 Å). Visualizations of models for all receptor cases are shown in Fig. S1 and Fig. S2. To see this figure in color, go online.

One of the most accurate predictions of ECL2 was obtained for two opioid receptors (DOR and MOR) and the CXCR4 receptor (see Fig. 2). For these receptors, all ECL2s formed two *β*-strands connected with a tight *β*-turn. Resulting loops resembled native-like conformations with high accuracy (see ECL2 prediction for the CXCR4 receptor, Fig. 3 *a*).

Our calculations reproduced the structure of ECL2 for two receptors (M2R and M3R) with good accuracy (RMSD^LC10^ = 3.70 Å and 3.89 Å, respectively). The lowest energy structure for ECL2 in the NTR1 receptor highly resembled its crystal structure; however, the entire loop fragment was tilted toward TMH4, resulting in high RMSD (8.82 Å). NTR1 was crystallized with bound peptide NTS interacting with ECL2 and ECL3. Ligand-receptor interaction may alter the structure and orientation of ECLs (all ligand-ECLs interactions present in the 13 receptor crystal structures used in this study are listed in Table S7). Note that ligand-receptor interactions were not taken into account during the modeling procedure, which may result in a different ECL2 orientation in the lowest energy models when compared to x-ray structures. The best NTR1 loop structure observed in the trajectory yielded low RMSD (2.99 Å). In the case of two adrenergic receptors (*β*1AR and *β*2AR) resulting loops also adopted native-like conformation and a short *α*-helix was formed as seen in crystal structures. Nevertheless, the position of the short *α*-helix deviated among the resulting models when compared to the crystal structures. The differences in the localization of the short *α*-helix were probably related to the high mobility of this long receptor loop (see Fig. 3 *b*).

The shortest predicted loop (13 residues) of the D3R receptor showed no secondary structure elements, resembling coil-like conformation. The predicted structure yielded RMSD^LE10^ = 2.88 Å and was in good agreement when compared to its crystal-derived form (see Fig. 3 *c*).

For S1PR, NOP, and RHO receptors our predictions were much less accurate and scoring methods (energy evaluation and structural clustering) were not able to point toward loop structures sufficiently resembling conformations of the crystal structures. The lowest RMSD values observed in the trajectory were equal to 4.4 Å, 4.47 Å, and 4.2 Å, respectively. In the case of RHO, more accurate prediction may require simulating the presence of the N-terminal domain, which was not accounted for in our calculations. The N-terminus provides additional stabilization for the ECL2 conformation as seen in crystal structure. Note that rhodopsin is a photoreceptor protein with a covalently bound ligand (retinal) buried deep in the binding site, whereas other GPCRs interact with diffusible ligands. When analyzing loop conformations we have to keep in mind a different role of ECL2 of rhodopsin, which forms a stable hydrophobic lid covering the binding site.

The longest predicted ECL2 (34 AA residues) for the A2AR receptor yielded RMSD^LE10^ = 5.88 Å. The predicted loop fragment (PRO:139 to ALA:165) differed from its native conformation by the absence of a short two-turn *α*-helix. The presence of the short helix was also not indicated in the input of secondary structure prediction (a coil type of secondary structure was assigned for the helix fragment, see Table S2). Therefore, in the A2AR case, a more accurate input of secondary structure may be helpful to generate models closer to the crystal structure. The remaining part of predicted ECL2 in A2AR (CYS:166 to VAL:172), in the vicinity of the ligand binding site, was in good agreement when compared to its crystal structure (one helical turn was created, see Fig. 3 *d*).

## Discussion

### Comparison with other structure prediction studies

In Table 3, we present comparison of our results to others in the literature. The comparison is based on two studies carried out by Goldfeld et al. (11) and Nikiforovich et al. (12). These studies, to the best of our knowledge, represent the most extensive and up-to-date reports concerning the restoration of ECL2 loops (performed for four GPCRs, as these were all crystallographically available GPCRs in 2009/2010 when those studies were carried out). We do not compare our results with ECL2 prediction made during homology modeling because the prediction of loops in homology models is a more difficult task than its restoration in crystal structures (56).

As shown in Table 3, our results are comparable to the other authors, except the lowest energy predictions (RMSD^LE^) of Goldfeld et al. (11) for *β*1AR, *β*2AR, and RHO receptors that matched the corresponding crystal structures with excellent RMSD values. It is worth emphasizing that our results and also those by Nikiforovich et al. (12) were obtained using a much less sophisticated modeling procedure (i.e., coarse-grained sampling combined with energy scoring that does not incorporate water or the lipid membrane). In our study, a single prediction took no longer than 0.5 h of single CPU time. More sophisticated methodologies (relying on a more precise system representation, like in the Goldfeld et al. (11) study) are computationally much more demanding. For instance, the prediction of the A2AR loop in the Goldfeld study took 145 days of single CPU time.

A direct comparison of the performance of GPCR loop modeling procedures is hampered by differences in the experimental data used in the calculations (see the discussion on the comparison of Goldfeld et al. (11) and Nikiforovich et al. (12) results in PNAS letters (54) and (57)). In the paragraphs below, we outline important details of our modeling procedure and differences between our calculations and others.

First, the definition of loop regions differs between studies. For cases presented in Table 3, we defined slightly shorter (typically by three residues) or slightly longer loop lengths (by two residues in the case of A2AR than Goldfeld et al. (11)). Similar differences in loop lengths exist between the Goldfeld et al. (11) and Nikiforovich et al. (12) studies. Because the differences are not large (compare Table 2 vs. Table 2 in (11) vs. Table VI in (12)), we believe they should not have any significant impact on prediction accuracy.

Second, for the A2AR case, in the Goldfeld et al. (11) and Nikiforovich et al. (12) studies, the calculations of RMSD values did not involve the ECL2 fragment between residues 149 and 155 (which is missing in the 3EML crystal structure used in the calculations); thus, only 27 residues were involved. On the contrary, we used a complete 34 residue fragment (from the 4EIY crystal structure); therefore, the RMSD comparison is not straightforward.

Third, our modeling procedure involved simulation of all EC loops (EC1, EC2, EC3) at the same time, using no knowledge of x-ray loop structure (except constraints on disulfide bridges). In contrast, in Goldfeld et al. (11), each single individual loop was obtained with the other loops fixed in their x-ray conformations.

Fourth, our modeling procedure used experimental distance restraints on disulfide bridges (DBs) (see also the CABS setup in the Methods section). In all receptor cases, we used knowledge about a well-conserved DB between TM3 and EC2 loops (being the only DB in five receptors) and about DBs within EC loops (a single one in seven receptors, and three DBs in A2AR, see the list of DBs in Table S1). In turn, Nikiforovich et al. (12) used in their modeling information about the conserved DB between TM3 and EC2 only (allowing DBs within EC loops to be predicted). However, they also repeated the calculations with inserted DBs in EC loops. The insertion did not result in significant changes in *β*1AR and *β*2AR and helped to improve prediction accuracy in A2AR (which was predicted with a similar RMSD^BEST^ value as in our calculations, see Table 3). In contrast, Goldfeld et al. (11) did not enforce experimental DBs (as explained in (57).); however, they used experimental atom-atom contact information within or between loops, derived from x-ray crystallography (Table S1 in (11)).

### Loop dynamics

In our modeling procedure, loop models are generated by the CABS model through a series of small local moves controlled by the Monte Carlo method. The long series of such moves was shown to accurately describe the realistic dynamics of globular proteins. Namely, CABS predictions of protein dynamics were shown to be consistent with experimental data (for the characterization of protein folding pathway dynamics (37–39)) and MD simulation data (for the characterization of near-native dynamics (35,36)).

**Figure 4:**
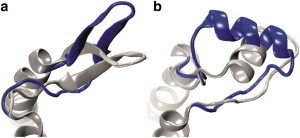
Models showing large-scale movements of the second extracellular loop. The lowest energy models of DOR (*a*) and *β*2AR (*b*) are presented in blue and superimposed on crystal structures shown in gray. Crystal structure fragments of transmembrane helices (TMH3 and TMH4) are also visualized. To see this figure in color, go online.

This work provides an ensemble view of ECL2 structures (in sets of 10 or 100 cluster representatives or the lowest energy models); however, it is only validated by comparison with x-ray structures frozen in a single conformational option. Analysis of the predicted ensembles suggests that, at least for some of the modeled receptors, ECL2s may be subjected to large molecular movements. For instance, the lowest energy models of *β*2AR and DOR have ECL2 structures very similar to those observed in crystal structures but significantly tilted. Namely, the short *α*-helix of *β*2AR is directed toward TMH4 and the *β*-sheet of DOR ECL2 is tilted toward TMH3 (see Fig. 4). To our knowledge, such large-scale loop rearrangements in GPCRs were found only by extremely long MD simulations (58) and by coarse-grained modeling (12). Considering the high flexibility of ECL2s (suggested but not precisely characterized by experiment) and its functional importance (8,15–23), future theoretical studies should aim at the characterization of an ensemble view of EC loops and its validation through experimental approaches.

## Conclusions

Previous reports showed that the CABS protein model offers state-of-the-art modeling capabilities, especially in difficult modeling cases (e.g., ab initio prediction of long protein fragments (24,26,27)). In this work, our goal was to test the ability of the CABS modeling approach to restore long loops (ECL2s) of 13 GPCRs (for all GPCRs with available crystal structures when this study was initiated). Based on the outcome of initial simulation runs, we introduced small modifications of the CABS algorithm that improved final performance. It should be noted that we used a low-cost computational procedure (coarse-grained CABS modeling that involves no membrane lipids, combined with a simple version of all-atom scoring and optimization). Despite the simplifications, our modeling approach yielded loop models of comparable accuracy as those obtained by other authors. Of importance, the results of our study provide benchmark data of newly crystallized GPCRs (other authors’ data were limited to the loop restoration of 4 or 5 GPCRs (11,12)) that enable researchers to compare their algorithms (our models are available from http://biocomp.chem.uw.edu.pl/GPCR-loop-modeling/).

Our modeling method provides a framework for the development of more sophisticated procedures. Future developments may include: incorporation of more accurate scoring (model quality assessment) methods, introduction of the lipid bilayer in CABS simulation (which may limit loop movements), use of sparse data from experiment (e.g., from GPCRRD database (59)) or theoretical predictions (e.g., residue-residue contact predictions), introduction of ligand presence, use of x-ray interpretations on flexibility of TM end positions, inclusion of more accurate secondary structure prediction tools, or extension of the method to use GPCRs homology models. Finally, the CABS-based approach offers promising perspectives for the simulation of long timescale conformational dynamics of ECLs in GPCRs.

## Figures and Tables

**Table 1:** Description of 13 GPCR receptor structures modeled in this study

Receptor name	PDB ID	Receptor description	Species
A2AR	4EIY	Adenosine receptor A2a	*Homo sapiens*
*β*1AR	2Y00	Beta-1 adrenergic receptor	*Meleagris gallopavo*
*β*2AR	2RH1	Beta-2 adrenergic receptor	*Homo sapiens*
M2R	3UON	Muscarinic acetylcholine receptor M2	*Homo sapiens*
M3R	4DAJ	Muscarinic acetylcholine receptor M3	*Rattus norvegicus*
CXCR4	3ODU	C-X-C chemokine receptor type 4	*Homo sapiens*
D3R	3PBL	Dopamine D3 receptor	*Homo sapiens*
NTR1	4GRV	Neurotensin receptor type 1	*Rattus norvegicus*
DOR	4EJ4	Delta-type opioid receptor	*Mus musculus*
NOP	4EA3	Nociceptin receptor	*Homo sapiens*
MOR	4DKL	Mu-type opioid receptor	*Mus musculus*
RHO	1U19	Rhodopsin	*Bos taurus*
S1PR	3V2W	Sphingosine 1-phosphate receptor 1	*Homo sapiens*

**Table 2:** ECLs restored in this study for 13 GPCRs

Receptor name	PDB ID	Loop	Loop sequence	Loop length	Residue numbering
A2AR	4EIY	ECL1	FCA	3	70–72
		ECL2	PMLGWNNCGQPKEGKNHSQGCGEGQVACLFEDVV	34	139–172
		ECL3	PDCSHA	6	260–265
*β*1AR	2Y00	ECL1	TWLW	4	105–110
		ECL2	WWRDEDPQALKCYQDPGCCDFVT	23	181–203
		ECL3	RDLV	4	317–320
*β*2AR	2RH1	ECL1	MWTF	4	98–101
		ECL2	WYRATHQEAINCYANETCCDFFT	23	173–195
		ECL3	DNLI	4	300–303
M2R	3UON	ECL1	YWPL	4	88–91
		ECL2	VRTVEDGECYIQFFS	15	168–182
		ECL3	APCI	4	414–417
M3R	4DAJ	ECL1	RWAL	4	132–135
		ECL2	KRTVPPGECFIQFLS	15	212–226
		ECL3	DSCI	4	517–520
CXCR4	3ODU	ECL1	NWYF	4	101–104
		ECL2	NVSEADDRYICDRFYP	16	176–191
		ECL3	IIKQ	4	269–272
D3R	3PBL	ECL1	GGVWNF	6	93–98
		ECL2	FNTTGDPTVCSIS	13	172–184
		ECL3	QTCHV	5	356–360
NTR1	4GRV	ECL1	HPWAF	5	133–137
		ECL2	GLQNRSGDGTHPGGLVCTPIV	21	209–229
		ECL3	DEQW	4	336–339
DOR	4EJ4	ECL1	TWPF	4	103–106
		ECL2	VTQPRDGAVVCMLQFPS	17	188–204
		ECL3	DINRR	5	288–292
MOR	4DKL	ECL1	TWPF	4	132–135
		ECL2	TTKYRQGSIDCTLTFSH	17	207–223
		ECL3	TIPE	4	307–310
NOP	4EA3	ECL1	FWPF	4	115–118
		ECL2	SAQVEDEEIECLVEIPT	17	190–206
		ECL3	VQPS	4	290–293
RHO	1U19	ECL1	YFVF	4	102–105
		ECL2	WSRYIPEGMQCSCGIDYYTPHEET	24	175–198
		ECL3	GSDF	4	280–283
S1PR	3V2W	ECL1	GATTYKL	7	106–112
		ECL2	WNCISALSSCSTVLPLY	17	182–198
		ECL3	KVKTCDILFR	10	283–292

**Table 3:** Comparison of our results to others in the literature

Receptor name (loop length)	Our data		Data of other authors		
	RMSD^BEST^ (Å)	RMSD^LE^ (Å)	RMSD^BEST^ (Å)	RMSD^LE^ (Å)	Reference, table, comments
A2AR (34)	4.7	5.9	5.9^a^	10.2^a^	(12), Table VI
			4.8^a^	4.8^a^	(12), Table VI, with inserted SS bonds
				4.4^a^	(11), Table 1
*β*1AR (23)	3.3	5.8	4.3	6.4	(12), Table VI
				1.6	(11), Table 1
*β*2AR (23)	3.4	5.5	3.8	7.4	(12), Table VI
				2.2	(11), Table 1
RHO (24)	4.2	8.2	4.7	8.4	(12), Table VI
				3.4	(11), Table 1

RMSDs (root mean-square deviation in Å to the crystal structure) for the second extracellular loop are listed: RMSD^BEST^ – representing the best model obtained and RMSD^LE^ – representing the lowest energy model. Our results are comparable to those of Nikiforovich et al. (12) and to those of Goldfeld et al. (11) in the case of A2Ar.

a Note that for the A2AR case we used crystal loop structure (PDB ID: 4EIY) of 34 residues for computing RMSD values, whereas the other authors used crystal structure (PDB ID: 3EML) of 27 residues in which 7 residues were missing.

## Supporting Material

Document S1. Two figures and seven tables

Document S1. Article plus Supporting Material
