# Supplementary material for: Structure Prediction of the Second Extracellular Loop in G-Protein-Coupled Receptors
Source: Biophys J. 2014 Jun 3;106(11):2408–16. doi: 10.1016/j.bpj.2014.04.022 (PMC4052351; doi:10.1016/j.bpj.2014.04.022)
Supplement: Document S1. Two figures and seven tables [file mmc1.pdf]

Structure Prediction of the Second Extracellular Loop in G-Protein-Coupled Receptors

Sebastian Kmiecik,[†] Michal Jamroz,[†] and Michal Kolinski[‡*]

[†]University of Warsaw, Faculty of Chemistry, Laboratory of Theory of Biopolymers, Pasteura 1, 02-093 Warsaw, Poland; and [‡]Mossakowski Medical Research Center, Polish Academy of Sciences, Bioinformatics Laboratory, Pawinskiego 5, 02-106 Warsaw, Poland

Supporting information

Figure S1. Predictions superimposed on crystal structures. Predicted models of the second extracellular loop are shown in red, together with the first and third extracellular loops colored in green, and reference crystal structure shown in gray. The models are presented for 7 receptor cases (the remaining are displayed in Figure S2): model with the lowest energy (LE), model showing the lowest RMSD from the 10 lowest energy models (LE10) and model showing the lowest RMSD from the 100 lowest energy models (LE100), model representing the largest cluster (LC), model showing the lowest RMSD from the representatives of 10 largest clusters (LC10), model showing the lowest RMSD from the representatives of 100 largest clusters (LC100).

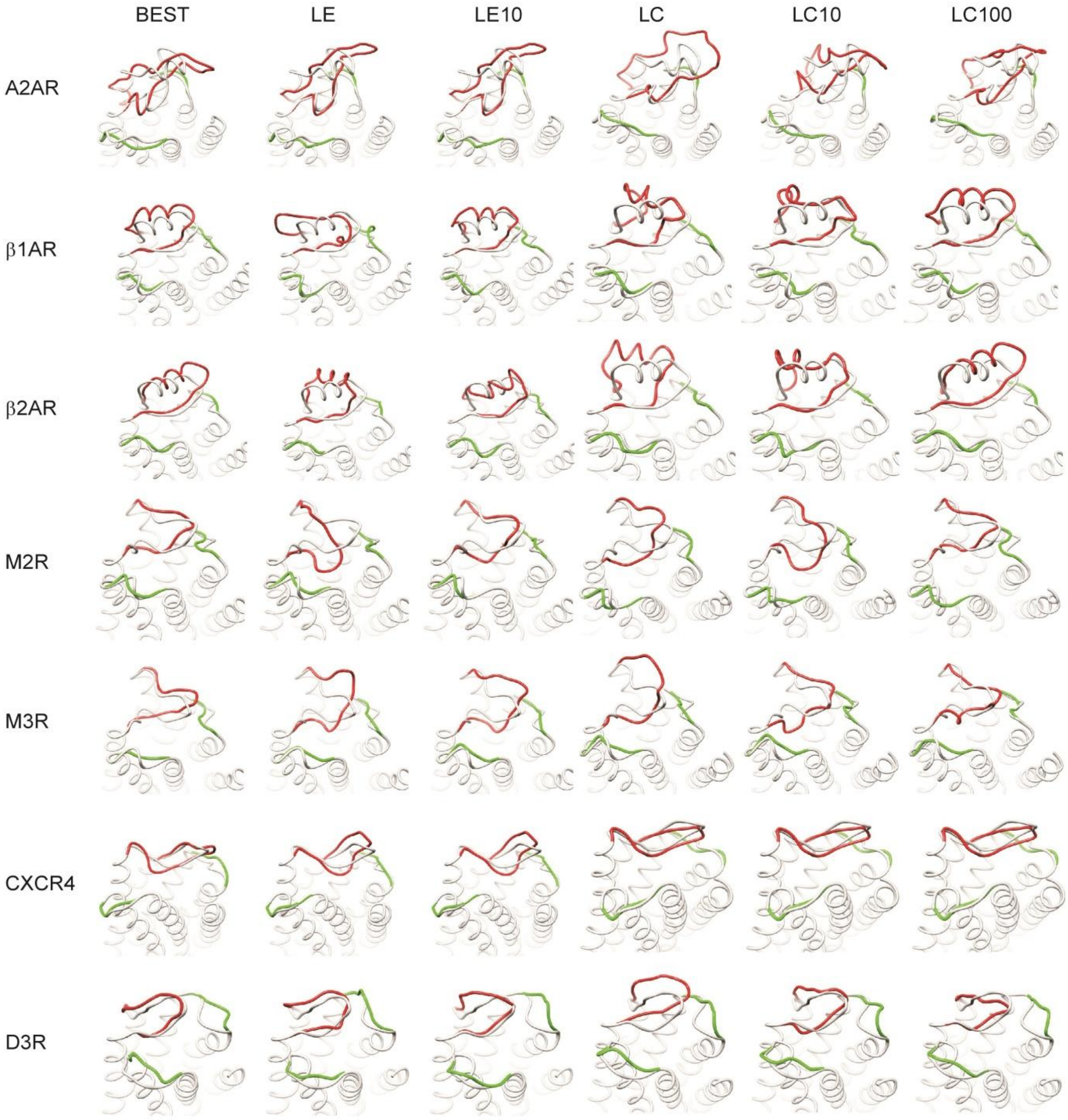

Figure S2. Predictions superimposed on crystal structures. Predicted models of the second extracellular loop are shown in red, together with the first and third extracellular loops colored in green, and the reference crystal structure shown in gray. The models are presented for 6 receptor cases (the remaining are displayed in Figure S1): model with the lowest energy (LE), model showing the lowest RMSD from the 10 lowest energy models (LE10) and model showing the lowest RMSD from the 100 lowest energy models (LE100), model representing the largest cluster (LC), model showing the lowest RMSD from the representatives of 10 largest clusters (LC10), model showing the lowest RMSD from the representatives of 100 largest clusters (LC100).

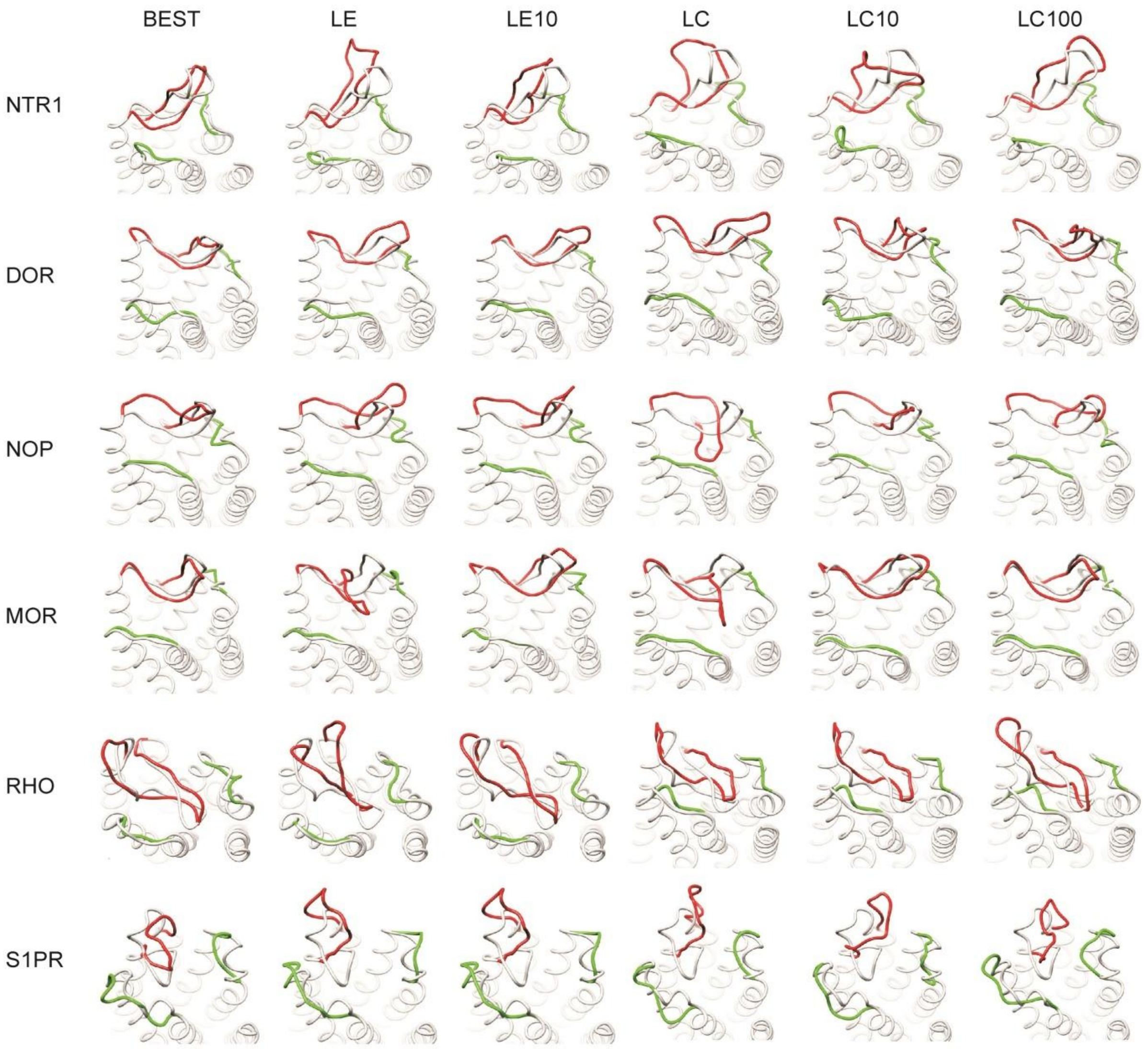

Table S1. Disulfide bridges used in the modeling. A well conserved disulfide bridge between TM3 and EC2 is marked bold. The other bridges occur within extracellular elements only.

**Receptor name**	**CYS-CYS pairs**
A2AR	71 ↔ 159, 74 ↔ 146, **77** ↔ **166**, 259 ↔ 262
β1AR	**114** ↔ **199**, 192 ↔ 198
β2AR	**106** ↔ **191**, 184 ↔ 190
M2R	**96** ↔ **176**, 413 ↔ 416
M3R	**140** ↔ **220**, 516 ↔ 519
CXCR4	28 ↔ 274, **109** ↔ **186**
D3R	**103** ↔ **181,** 355 ↔ 358
NTR1	**142** ↔ **225**
DOR	**121** ↔ **198**
NOP	**123** ↔ **200**
MOR	**140** ↔ **217**
RHO	**110** ↔**187**
S1PR	**184** ↔ **191**, 282 ↔ 287

Table S2. Consensus prediction and DSSP assignment of secondary structure for extracellular loops.

Receptor name	Secondary structure of ECL2	
A2AR	AA sequence<br>consensus SS prediction<br>DSSP SS assignment	PMLGWNNCGQPKEGKNHSQGCGEGQVACLFEDVV<br>CCCCCCCCCCCCCCCCCCCCCCCCEEEEEEECCC<br>HHHCCCCCCCCCHHHHHHHCCCCCEEECCHHHHC
β1AR	AA sequence<br>consensus SS prediction<br>DSSP SS assignment	WWRDEDPQALKCYQDPGCCDFVT<br>CCCCCCHHHHHHCCCCCCCCCCC<br>CCCCCCHHHHHHHHCCCCCCCCC
β2AR	AA sequence<br>consensus SS prediction<br>DSSP SS assignment	WYRATHQEAINCYANETCCDFFT<br>CCCCCHHHHHHHHCCCCCCCCCC<br>CCCCCCHHHHHHHHCCCCCCCCC
M2R	AA sequence<br>consensus SS prediction<br>DSSP SS assignment	VRTVEDGECYIQFFS<br>CEEEECCEEEEHHHH<br>CCCCCCCCCCCCCCC
M3R	AA sequence<br>consensus SS prediction<br>DSSP SS assignment	KRTVPPGECFIQFLS<br>CEEEECCEEEEHHHH<br>CCCCCCCCCCCCCCC
CXCR4	AA sequence<br>consensus SS prediction<br>DSSP SS assignment	NVSEADDRYICDRFYP<br>CCCCCCEEEEECCCCC<br>EEEEECCEEEEEECCC
D3R	AA sequence<br>consensus SS prediction<br>DSSP SS assignment	FNTTGDPTVCSIS<br>CCCCCCCEEEEEC<br>CCCCCCCCCCCCC
NTR1	AA sequence<br>consensus SS prediction<br>DSSP SS assignment	GLQNRSGDGTHPGGLVCTPIV<br>CCCCCCCCCCCCCCEEEEECC<br>EEEECCCCCCCCCCEEEEECC
DOR	AA sequence<br>consensus SS prediction<br>DSSP SS assignment	VTQPRDGAVVCMLQFPS<br>CCCCCCCEEEEEEECCC<br>EEEEECCEEEEEECCCC
MOR	AA sequence<br>consensus SS prediction<br>DSSP SS assignment	TTKYRQGSIDCTLTFSH<br>CCCCCCCCEEEEEEEEC<br>CCEECCCCEECCCCCCC
NOP	AA sequence<br>consensus SS prediction<br>DSSP SS assignment	SAQVEDEEIECLVEIPT<br>CCCCCCCCEEEEEEECC<br>EECCCCCCCCEEECCCC
RHO	AA sequence<br>consensus SS prediction<br>DSSP SS assignment	WSRYIPEGMQCSCGIDYYTPHEET<br>CCCCCCCCEEEEECCCEEECCCCC<br>CCCEEEECCCCEEEECCCCCCCCC
S1PR	AA sequence<br>consensus SS prediction<br>DSSP SS assignment	WNCISALSSCSTVLPLY<br>CCHHHHCCCCCCEEECC<br>CCCCCCCCCCCCCCCCC

E - extended conformation, H - helix, C - undefined/coil

Table S3. Results of scoring according to all-atom energy values. RMSD values between backbone atoms of three extracellular loops and their native counterparts are shown.

Receptor name	Loop	$RMSD^{BEST}$ (Å)	$RMSD^{LE}$ (Å)	$RMSD^{LE10}$ (Å)	$RMSD^{LE100}$ (Å)
A2AR	ECL1	0.40	1.15	1.15	1.15
	ECL2	4.67	5.88	5.88	5.88
	ECL3	0.70	2.50	2.50	2.50
	ECL1+ECL2+ECL3	4.24	5.32	5.32	5.32
β1AR	ECL1	1.43	2.47	3.39	3.14
	ECL2	3.28	5.81	3.57	3.40
	ECL3	0.65	1.01	2.24	2.01
	ECL1+ECL2+ECL3	3.07	5.09	3.40	3.22
β2AR	ECL1	0.90	2.27	2.83	2.08
	ECL2	3.38	5.51	4.43	3.82
	ECL3	0.76	2.21	2.33	1.36
	ECL1+ECL2+ECL3	3.07	4.88	4.04	3.41
M2R	ECL1	0.62	1.05	1.91	1.91
	ECL2	2.65	5.12	2.87	2.87
	ECL3	0.43	1.81	1.20	1.20
	ECL1+ECL2+ECL3	2.50	4.23	2.50	2.50
M3R	ECL1	0.76	3.57	3.15	1.99
	ECL2	2.53	4.65	4.11	2.98
	ECL3	0.51	2.60	2.17	2.00
	ECL1+ECL2+ECL3	2.46	4.18	3.68	2.68
CXCR4	ECL1	1.45	2.73	2.73	3.44
	ECL2	2.41	3.08	3.08	2.41
	ECL3	0.41	0.77	0.77	1.10
	ECL1+ECL2+ECL3	2.45	2.77	2.77	2.46
D3R	ECL1	1.08	3.47	5.04	4.00
	ECL2	2.14	3.43	2.88	2.36
	ECL3	1.06	2.73	2.95	2.63
	ECL1+ECL2+ECL3	2.44	3.30	3.56	2.91
NTR1	ECL1	1.90	3.59	2.89	3.23
	ECL2	2.99	8.82	4.74	3.70
	ECL3	0.81	1.77	1.94	2.03
	ECL1+ECL2+ECL3	2.90	7.55	4.20	3.45
DOR	ECL1	1.20	2.41	2.23	2.68
	ECL2	1.92	5.04	3.63	2.93
	ECL3	1.43	3.83	3.68	1.87
	ECL1+ECL2+ECL3	2.15	4.51	3.46	2.72
MOR	ECL1	0.79	2.87	2.31	2.55
	ECL2	1.92	6.78	3.22	2.43
	ECL3	0.55	0.94	1.01	1.36
	ECL1+ECL2+ECL3	2.08	5.72	2.84	2.31
NOP	ECL1	0.78	3.06	2.89	3.51
	ECL2	4.47	6.22	5.48	4.55
	ECL3	0.58	2.83	1.80	1.86
	ECL1+ECL2+ECL3	4.05	5.40	4.72	4.07
RHO	ECL1	1.03	4.16	1.49	3.62
	ECL2	4.20	8.21	4.68	4.31
	ECL3	0.56	1.43	1.59	2.65
	ECL1+ECL2+ECL3	3.90	7.28	4.13	4.06
S1PR	ECL1	1.63	3.39	3.39	4.23
	ECL2	4.40	7.40	7.40	6.86
	ECL3	2.04	4.90	4.90	6.16
	ECL1+ECL2+ECL3	4.18	6.07	6.07	6.20

$RMSD^{BEST}$ – lowest RMSD observed in all CABS generated models
$RMSD^{LE}$ – RMSD value for the loop corresponding to the model with the lowest potential energy

$RMSD^{LE10}$ – lowest RMSD value for ECL2 observed among 10 models with the lowest potential energy and corresponding RMSD values for ECL1, ECL3 and combination of ECL1+ECL2+ECL3
$RMSD^{LE100}$ – lowest RMSD values for ECL2 observed among 100 models with the lowest potential energy and corresponding RMSD values for ECL1, ECL3 and combination of ECL1+ECL2+ECL3

Table S4. Results of scoring according to structural clustering. RMSD values between backbone atoms of three extracellular loops and their native counterparts are presented.

Receptor name	Loop	$RMSD^{BEST}$ (Å)	$RMSD^{LC}$ (Å)	$RMSD^{LC10}$ (Å)	$RMSD^{LC100}$ (Å)
A2AR	ECL1	0.40	1.22	1.32	1.66
	ECL2	4.67	8.74	5.00	5.85
	ECL3	0.70	2.29	1.55	2.31
	ECL1+ECL2+ECL3	4.24	7.83	4.50	5.29
β1AR	ECL1	1.43	3.37	3.55	2.42
	ECL2	3.28	6.85	5.33	3.28
	ECL3	0.65	2.18	1.92	2.34
	ECL1+ECL2+ECL3	3.07	6.07	4.81	3.07
β2AR	ECL1	0.90	2.42	2.26	2.45
	ECL2	3.38	6.73	4.41	3.38
	ECL3	0.76	2.95	3.45	2.51
	ECL1+ECL2+ECL3	3.07	5.96	4.08	3.17
M2R	ECL1	0.62	2.22	2.42	2.81
	ECL2	2.65	4.13	3.70	2.65
	ECL3	0.43	1.39	1.65	1.59
	ECL1+ECL2+ECL3	2.50	3.51	3.23	2.53
M3R	ECL1	0.76	2.56	2.26	2.27
	ECL2	2.53	4.64	3.89	2.63
	ECL3	0.51	1.86	1.80	2.24
	ECL1+ECL2+ECL3	2.46	3.97	3.36	2.50
CXCR4	ECL1	1.45	3.35	3.35	3.35
	ECL2	2.41	3.56	3.56	3.56
	ECL3	0.41	1.02	1.02	1.02
	ECL1+ECL2+ECL3	2.45	3.24	3.24	3.24
D3R	ECL1	1.08	3.66	3.80	4.00
	ECL2	2.14	6.10	2.57	2.36
	ECL3	1.06	3.18	3.19	2.63
	ECL1+ECL2+ECL3	2.44	5.06	3.05	2.91
NTR1	ECL1	1.90	3.27	3.23	3.23
	ECL2	2.99	7.84	4.82	3.70
	ECL3	0.81	3.05	2.85	2.03
	ECL1+ECL2+ECL3	2.90	6.79	4.37	3.45
DOR	ECL1	1.20	1.90	2.91	2.84
	ECL2	1.92	4.88	3.24	2.08
	ECL3	1.43	2.62	4.31	3.06
	ECL1+ECL2+ECL3	2.15	4.18	3.42	2.42
MOR	ECL1	0.79	2.31	3.04	2.42
	ECL2	1.92	6.96	2.24	1.92
	ECL3	0.55	2.08	1.85	2.32
	ECL1+ECL2+ECL3	2.08	5.87	2.33	2.08
NOP	ECL1	0.78	3.18	3.09	3.83
	ECL2	4.47	9.33	5.80	5.56
	ECL3	0.58	1.78	2.13	1.84
	ECL1+ECL2+ECL3	4.05	7.83	5.01	4.89
RHO	ECL1	1.03	4.21	4.21	2.48
	ECL2	4.20	5.11	5.11	4.36
	ECL3	0.56	2.98	2.98	4.11
	ECL1+ECL2+ECL3	3.90	4.79	4.79	4.14
S1PR	ECL1	1.63	3.39	2.69	5.32
	ECL2	4.40	8.49	7.66	5.85
	ECL3	2.04	3.58	4.03	3.51
	ECL1+ECL2+ECL3	4.18	6.50	5.96	5.15

$RMSD^{BEST}$ – the lowest RMSD observed in all CABS generated models

$RMSD^{LC}$ – RMSD value for the loop corresponding to the representative model of the largest cluster from CABS simulations

$RMSD^{LC10}$ – lowest RMSD values for ECL2 observed among 10 models representing the 10 largest clusters and corresponding RMSD values for ECL1, ECL3 and combination of ECL1+ECL2+ECL3
$RMSD^{LC100}$ – lowest RMSD values for ECL2 observed among 100 models representing the 100 largest clusters and corresponding RMSD values for ECL1, ECL3 and combination of ECL1+ECL2+ECL3

Table S5. Comparison of ECL2 structures of receptors used in the modeling with other available x-ray structures. ECL2 are compared using RMSD measure for ECL2 backbone atoms. RMSDs values were calculated after optimal superimposition of compared models with excluded loop fragments. X-ray structures were taken from GPCRSD database (structures available by March 12, 2014 - Jianyi Yang and Yang Zhang. GPCRSD: a database for experimentally solved GPCR structures: http://zhanglab.ccmb.med.umich.edu/GPCRSD/).

Receptor name	PDB ID of structure used in the modeling	PDB ID (of alternative X-ray structure): RMSD (in Angstroms, to structure used in the modeling)
RHO	1U19	1F88: 0.48, 1GZM: 0.34, 1HZX: 0.42, 1L9H: 0.34, 2G87: 0.26, 2HPY: 0.36, 2I35: 0.44, 2I36: 0.92, 2I37: 0.93, 2J4Y: 0.73, 2PED: 0.29, 2X72: 1.93, 3C9L: 0.34, 3C9M: 0.67, 3CAP: 1.75, 3DQB: 1.91, 3OAX: 0.28, 3PQR: 1.85, 3PXO: 1.84, 4A4M: 1.93, 4BEY: 1.87, 4BEZ: 1.88, 4J4: Q1.86
β1AR	2Y00	2VT4: 0.67, 2Y01: 0.84, 2Y02: 0.59, 2Y03: 0.62, 2Y04: 0.74, 2YCW: 0.60, 2YCX: 0.82, 2YCY: 0.78, 2YCZ: 0.95, 3ZPQ: 0.52, 3ZPR: 0.41, 4AMI: 0.92, 4AMJ: 0.41, 4GPO: 0.84
A2AR	4EIY	2YDO: 2.14, 2YDV: 2.30, 3EML: 0.42, 3PWH: 0.58, 3QAK: 0.69, 3REY: 0.58, 3RFM: 0.49, 3UZA: 0.52, 3UZC: 0.49, 3VG9: 1.27, 3VGA: 0.70
β2AR	2RH1	3D4S: 0.46, 3NY8: 0.57, 3NY9: 0.59, 3NYA: 0.53, 3P0G: 0.71, 3PDS: 0.83, 3SN6: 0.89, 4GBR: 0.43, 4LDE: 0.76, 4LDL: 0.74, 4LDO: 0.69
CXCR4	3ODU	3OE0: 1.31, 3OE6: 0.68, 3OE8: 0.86, 3OE9: 1.91
NTR1	4GRV	3ZEV: 1.46, 4BUO: 1.54, 4BV0: 1.62, 4BWB: 1.29
M2R	3UON	4MQS: 2.46, 4MQT: 2.50
DOR	4EJ4	4N6H: 0.85
S1PR	3V2W	3V2Y: 0.53

The RMSD values calculated for ECL2 are low (RMSD < 1 Å) when comparing receptor-antagonist complexes. Higher RMSD values (RMSD < 2.5 Å) were observed when comparing receptor-antagonist complexes and receptors with bound agonists, e.g. comparing PDB ID:3UON (M2R with a bound antagonist) to PDB ID: 4MQS (M2R with a bound agonist) yields RMSD = 2.46 Å. Please note that superposition of two receptor structures corresponding to inactive and active conformation is ambiguous due to significant changes in the position of TM helices. Therefore the measure of loop displacement is not exact. Visual inspection of different receptor structures indicated very small differences in ECL2 structure among the same receptor subtypes.

Table S6. Comparison of the predicted models to available x-ray structures. X–ray structures used in the calculations are listed in Table S5. ECL2 are compared using RMSD measure for ECL2 backbone atoms. RMSDs values were calculated after optimal superimposition of compared models with excluded loop fragments. For each receptor the following values are provided: average RMSD for available x-ray structures (together with standard deviation), minimum and maximum difference between RMSD calculated for receptor structure used in the modeling (values reported in the manuscript) and RMSD calculated for other available x-ray structure.

Receptor name	Predicted model	Average RMSD (standard deviation)	RMSD difference (minimum, maximum)
RHO	BEST	4.42 (±0.34)	-0.26, 0.85
	LC100	4.56 (±0.29)	-0.08, 0.77
	LC10	5.25 (±0.23)	-0.15, 0.63
	LC	5.25 (±0.23)	-0.15, 0.63
	LE100	4.54 (±0.31)	-0.13, 0.76
	LE10	4.85 (±0.26)	-0.14, 0.69
	LE	8.10 (±0.12)	-0.29, 0.08
β1AR	BEST	3.22 (±0.16)	-0.34, 0.16
	LC100	3.22 (±0.16)	-0.34, 0.16
	LC10	5.33 (±0.09)	-0.17, 0.15
	LC	6.85 (±0.08)	-0.10, 0.16
	LE100	3.45 (±0.08)	-0.14, 0.25
	LE10	3.49 (±0.20)	-0.40, 0.26
	LE	5.75 (±0.13)	-0.32, 0.10
A2AR	BEST	4.62 (±0.17)	-0.41, 0.18
	LC100	5.78 (±0.08)	-0.27, 0.05
	LC10	5.14 (±0.24)	-0.10, 0.67
	LC	8.75 (±0.08)	-0.07, 0.21
	LE100	5.96 (±0.13)	-0.05, 0.48
	LE10	5.96 (±0.13)	-0.05, 0.48
	LE	5.96 (±0.13)	-0.05, 0.48
β2AR	BEST	3.35 (±0.12)	-0.17, 0.29
	LC100	3.35 (±0.12)	-0.17, 0.29
	LC10	4.43 (±0.05)	-0.07, 0.13
	LC	6.75 (±0.22)	-0.31, 0.52
	LE100	3.84 (±0.13)	-0.12, 0.37
	LE10	4.41 (±0.08)	-0.11, 0.20
	LE	5.59 (±0.10)	-0.02, 0.27
CXCR4	BEST	2.51 (±0.08)	0, 0.25
	LC100	3.72 (±0.14)	0, 0.37
	LC10	3.72 (±0.14)	0, 0.37
	LC	3.72 (±0.14)	0, 0.37
	LE100	2.51 (±0.08)	0, 0.25
	LE10	2.94 (±0.15)	-0.42, 0
	LE	2.94 (±0.15)	-0.42, 0
NTR1	BEST	3.06 (±0.04)	0, 0.11
	LC100	3.55 (±0.08)	-0.25, 0
	LC10	4.75 (±0.06)	-0.15, 0
	LC	7.34 (±0.25)	-0.65, 0
	LE100	3.55 (±0.08)	-0.25, 0
	LE10	5.14 (±0.23)	0, 0.63
	LE	8.24 (±0.29)	-0.75, 0
M2R	BEST	3.61 (±0.68)	0, 1.47
	LC100	3.61 (±0.68)	0, 1.47
	LC10	4.08 (±0.28)	0, 0.64
	LC	4.55 (±0.30)	0, 0.66
	LE100	3.42 (±0.39)	0, 0.86
	LE10	3.42 (±0.39)	0, 0.86
	LE	5.10 (±0.04)	-0.08, 0.02
DOR	BEST	1.95 (±0.03)	0, 0.06
	LC100	2.16 (±0.07)	0, 0.15
	LC10	3.24 (±0.00)	-0.01, 0
	LC	4.75 (±0.12)	-0.25, 0
	LE100	2.89 (±0.04)	-0.08, 0
	LE10	3.48 (±0.14)	-0.29, 0
	LE	4.94 (±0.10)	-0.20, 0

S1PR	BEST	4.47 (±0.08)	0, 0.15
	LC100	5.91 (±0.06)	0, 0.12
	LC10	7.75 (±0.10)	0, 0.19
	LC	8.55 (±0.07)	0, 0.13
	LE100	6.87 (±0.01)	0, 0.01
	LE10	7.46 (±0.06)	0, 0.12
	LE	7.46 (±0.06)	0, 0.12

Table S7. Residues located within ECLs fragments and interacting with bound ligands as seen in x-ray structures used in this study.

Receptor name	PDB ID	Ligand	ECL:(residues)
A2AR	4EIY	UK-432097	ECL2:(Phe168, Glu169)
β1AR	2Y00	Dobutamine	ECL2:(Phe201)
β2AR	2RH1	Carazolol	ECL2:(Phe193)
M2R	3UON	QNB	ECL2:(Phe181)
M3R	4DAJ	Tiotropium	-
CXCR4	3ODU	IT1t	-
D3R	3PBL	Eticlopride	-
NTR1	4GRV	NTS	ECL2:(Arg213, Val224, Thr226), ECL3:(Glu337, Trp339)
DOR	4EJ4	Naltrindole	-
NOP	4EA3	C-24	-
MOR	4DKL	β-FNA	-
RHO	1U19	Retinal	-
S1PR	3V2W	ML056	-
